# Predictors of Hemorrhage and Re-Intervention in Renal Angiomyolipoma Following Transcatheter Arterial Embolization

**DOI:** 10.3390/jcm14196990

**Published:** 2025-10-02

**Authors:** Abinaya Ramakrishnan, David Reilly, James Sayre, Parsa Asachi, Kameel Khabaz, Matthew Quirk, Adam Plotnik, Antoinette Gomes, Siddharth A. Padia, Justin P. McWilliams

**Affiliations:** 1 Department of Radiology, Division of Interventional Radiology, UCLA Medical Center, Los Angeles, CA 90095, USA; aramakrishnan@mednet.ucla.edu (A.R.); dreilly@mednet.ucla.edu (D.R.); jsayre@mednet.ucla.edu (J.S.); kkhabaz@mednet.ucla.edu (K.K.); mquirk@mednet.ucla.edu (M.Q.); aplotnik@mednet.ucla.edu (A.P.); agomes@mednet.ucla.edu (A.G.); spadia@mednet.ucla.edu (S.A.P.); 2 Department of Internal Medicine, NYU Grossman School of Medicine, New York 10016 NY, USA; pasachi@mednet.ucla.edu

**Keywords:** AML, angiomyolipoma, hemorrhage, embolization

## Abstract

**Purpose:** Renal angiomyolipomas (AMLs) are benign renal neoplasms that may lead to spontaneous hemorrhage. Transcatheter arterial embolization (TAE) is a nephron-sparing treatment option, yet data on predictors of hemorrhage and re-intervention remain limited. This study evaluates clinical and radiologic outcomes of TAE and identifies predictors of hemorrhage and repeat embolization. **Materials and Methods**: A retrospective review of 66 patients (69 AMLs) undergoing TAE between 2010 and 2024 was conducted. Clinical, radiological, and procedural variables were analyzed. Tumor size, vascularity, and aneurysmal features were assessed pre- and post-embolization. Logistic regression models identified predictors of hemorrhage and repeat TAE. **Results:** Pre-treatment tumor diameter was the only significant predictor of hemorrhage (*p* = 0.011), with a threshold of 6.8 cm yielding 84.6% sensitivity and 71.3% specificity. All hemorrhagic tumors measured ≥4 cm. Post-embolization tumor volume predicted repeat TAE (*p* = 0.001), with a 248 mL cutoff. TAE significantly reduced tumor diameter (−33.5%) and volume (−60%) (*p* < 0.001). Radiologic success was achieved in 97% of cases, with a durable success rate of 84%. Clinical success was 94%, and complications occurred in 7.2% of patients, including two major events. **Conclusions:** TAE is a safe and effective treatment for renal AMLs. Tumor diameter >6.8 cm is a strong predictor of hemorrhage, while larger post-embolization volumes predict the need for re-intervention. These findings challenge the conventional 4 cm treatment threshold and support more individualized management strategies incorporating tumor morphology and response to embolization.

## 1. Introduction

Renal angiomyolipomas (AMLs) are benign neoplasms composed of mature adipose tissue, smooth muscle, and dysmorphic blood vessels. They are identified in approximately 0.1% to 2.2% of the adult population, with a higher prevalence in females, and are most commonly sporadic in nature [1,2,3,4,5,6]. A small proportion of cases are associated with genetic conditions such as tuberous sclerosis complex (TSC) and lymphangioleiomyomatosis (LAM) [5,6]. Although most AMLs remain asymptomatic, they can occasionally present with symptoms such as abdominal or flank pain, hematuria, hypertension, anemia, or acute retroperitoneal hemorrhage. Factors associated with an increased risk of hemorrhage include larger tumor size, multifocality, and vascular abnormalities [7,8,9].

The conventional threshold for predicting hemorrhagic risk in AMLs originated from a 1986 study by Oesterling et al., which proposed a tumor size of 4 cm as a critical marker for clinical management [10]. However, more recent studies have challenged the validity of this criterion, noting that only about 30% of renal AMLs exceeding 4 cm become symptomatic [11]. Emerging evidence indicates that factors such as tumor vascularity and the presence or size of intralesional aneurysms may be more accurate predictors of hemorrhagic risk than size alone [12]. Additionally, the proportion of macroscopic fat content may play a role, as fat-poor AMLs are thought to bleed more readily due to the higher proportion of abnormal vascular structures [13]. Further research is warranted to refine risk stratification and optimize treatment strategies aimed at preventing hemorrhage.

Current management options for symptomatic or large (>4 cm) AMLs include radical or partial nephrectomy, transcatheter arterial embolization (TAE), percutaneous ablation, and, in patients with tuberous sclerosis complex (TSC), systemic therapy with a mammalian target of rapamycin (mTOR) inhibitor such as everolimus [5,6]. In recent years, TAE has gained favor as a first-line treatment for bleeding AMLs in emergency settings and is used prophylactically for larger tumors [14,15]. TAE offers a nephron-sparing approach with fewer complications compared to surgical options, though repeat interventions may be necessary due to residual symptoms or recurrence [16,17,18].

Despite the growing use of transcatheter arterial embolization (TAE) as a therapeutic approach for AMLs, limited data exist on predictors of tumor response and risk of recurrent hemorrhage following treatment. The purpose of our study was to evaluate predictors of tumoral hemorrhage and to assess tumor characteristics following embolization in relation to the risk of rebleeding.

## 2. Methods

This study was approved by the local institutional review board. Clinical data of all patients between 1 January 2010 and 31 December 2024 who underwent TAE for renal AML were obtained retrospectively. Data collected included patient demographics, size of AML, vascularity, presence/size of intralesional aneurysm, renal function, diagnosis of neurocutaneous disorder, elective or emergency procedure, length of stay, embolic agent(s) used, perioperative complications, and further AML treatments. Indications for TAE included prophylactic embolization of incidentally detected tumors > 4 cm in maximum diameter (42, 61%), symptomatic tumors (13, 19%), and for acute hemorrhage (14, 20%) (Table 1). TAE was performed using a variety of embolic agents, including particles, coils, and sclerosing agents (Table 2).

Tumor characteristics were assessed on cross-sectional imaging (CT and MRI) at two main time points: pre-embolization and post-embolization at the end of the follow-up period. The size and vascularity were identified in initial radiographic imaging. Size (diameter) was measured in the longest axis in the transverse plane prior to treatment and in that same axis afterwards. Volume was calculated using the ellipsoid formula (transverse diameter × craniocaudal diameter × anteroposterior diameter × π/6). Tumor vascularity was estimated into quartile ranges (%) based on the proportion of enhancement, or soft tissue component in the absence of contrast administration, by two concurring board-certified radiologists (JPM and DR). The presence of an aneurysm or arteriovenous malformation was captured by both CT and angiographic images.

Complications were recorded and classified as per the Society of Interventional Radiology Adverse Event classification system [19]. Technical success was defined as complete selective embolization without any procedure-related complications within 24 h. Clinical success was defined as the lack of complications and symptoms in the follow-up period after TAE. Radiological success was subcategorized into reduction in size and reduction in vascularity during the follow-up period after one episode of TAE. Durable radiological success is defined as a sustained reduction in tumor size and vascularity during the follow-up period and absence of bleeding after a single session of TAE.

Data were collected via the electronic medical record (Epic) and the picture archiving and communication system with Centricity Picture Archiving and Communication System (PACS; GE Healthcare, Chicago, IL, USA) and Visage Imaging PACS (Visage Imaging, Sydney, Australia). Baseline characteristics were compared using a two-sample *t*-test or Mann–Whitney tests for continuous data or Fisher’s exact tests for categorical variables. A stepwise multivariable logistic regression analysis was performed at the time of embolization to identify predictors of hemorrhage, including age, gender, etiology, angiographic features, tumor location, vascularity, tumor diameter, and volume. A receiver operating characteristic (ROC) analysis was performed using this logistic model, yielding an area under the curve (AUC) of 0.644 (95% CI 0.4634–0.8254). Another multivariable logistic regression model was created to identify predictors of repeat TAE. All statistical tests were two-sided, and differences were considered statistically significant when *p* < 0.05. Statistical evaluation was conducted using R software v.4.4.2 (R Foundation; www.r-project.org).

## 3. Results

### 3.1. Demographics

**Figure 1:**
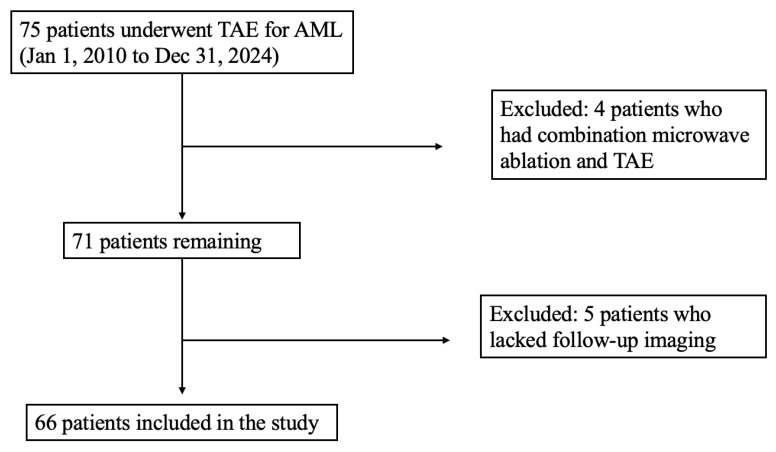
Inclusion criteria.

Sixty-six patients were treated for AML during the study period, and a total of 69 primary TAEs were performed as described in Figure 1. AMLs were found more frequently in females (52, 79%) than males (14, 21%), with an average age at time of embolization of 55 ± 15.8 years. Most lesions were exophytic (61, 88%). A minority had aneurysmal vessels identified on CT or angiography (29, 42%). Tumors were predominantly sporadic in nature (54, 78%) with a smaller subset attributed to TSC/LAM (15, 22%). Additional tumor characteristics are summarized in Table 1.

Fourteen patients (21%) presented with at least one episode of hemorrhage prior to TAE, while the remaining cases underwent prophylactic embolization due to tumor size, symptoms, or underlying TSC/LAM. Among those in the hemorrhage group, seven patients presented acutely without a prior AML diagnosis, whereas the other seven had known AMLs but had not previously undergone embolization, as they did not meet clinical or radiographic criteria at the time of their last imaging.

### 3.2. Size and Vascularity

The average tumor diameter of AML pre-embolization was 7.5 ± 3.8 cm, and the average volume was 209 ± 315 mL (Table 3). Among the treated AMLs, 62 tumors measured ≥4.0 cm, 36 were ≥6.0 cm, and 24 were ≥8.0 cm. Of the seven tumors measuring below 4 cm, two were embolized prophylactically due to growth and near-threshold size (3.9 cm), one was treated because the patient had an additional tumor measuring 4.5 cm, two were treated due to pain, and one was treated due to known TSC. On pre-embolization imaging, 39 tumors demonstrated >50% vascular composition, with 21 tumors between 76 and 100%.

Embolization was performed with a variety of primary embolics: alcohol with or without lipiodol (25), particles alone (17), a combination of alcohol and particles (13), alcohol and liquid embolic (12), or liquid embolic alone (2). Twelve lesions were treated with supplementary coils (Table 2). Following TAE, there was a significant decrease in tumor diameter and volume (*p* < 0.001). The average tumor diameter decreased by 33.5 ± 23.7%, and the average volume was reduced by 60 ± 48.0% (Table 3). Most AMLs post-embolization demonstrated a marked reduction in vascularity, with 53 lesions (75%) showing <25% vascular composition, and none exceeding 75% vascularity. The decrease in tumor vascularity following TAE was statistically significant (*p* < 0.001). Three AMLs remained hypervascular (50–75%) after embolization, one patient required repeat embolization and subsequent partial nephrectomy, while the other two patients did not undergo further embolization or surgery.

After categorizing patients into hemorrhage (n = 14) and non-hemorrhage (n = 55) groups based on their initial presentation, all tumors in the hemorrhage group measured ≥4.0 cm; 10 were ≥6.0 cm, 8 were ≥8.0 cm, and 5 were ≥10.0 cm. Tumors in the hemorrhage group exhibited larger diameters (8.4 ± 4.4 cm vs. 7.2 ± 3.5 cm, *p* = 0.085) and volumes (414 ± 520 mL vs. 156 ± 214 mL, *p* = 0.053) prior to embolization, though these differences were not statistically significant. The overall reduction in tumor diameter (−3.2 ± 1.7 cm vs. −2.3 ± 2.5 cm, *p* = 0.034) and volume (−221 ± 251 mL vs. −89 ± 140 mL, *p* = 0.016) was significantly greater in the hemorrhage group. Vascular composition was similar between groups both before (*p* = 0.987) and after (*p* = 0.506) embolization (Table 4).

### 3.3. Re-Intervention

The average clinical follow-up duration was 39.6 ± 34.4 months, and the average radiological follow-up was 25.3 ± 25.6 months. Five patients lacked sufficient imaging follow-up data for review and were excluded from the study. Nine patients underwent repeat TAE. Three patients required repeat TAE for hemorrhage, and another three patients due to increased vascularity: two patients showed progression from 25–50% to 50–75% vascular composition following initial embolization, and the other had persistent hypervascularity (50–75%). Another two patients required repeat embolization due to tumor growth during follow-up—one from 2.9 cm to 4.0 cm, and the other from 5.6 cm to 8.9 cm. One patient required repeat embolization for symptom management. Based on these outcomes, the clinical success rate was 94.0% (65/69). In terms of immediate radiological success, 97% (67/69) of tumors had a reduction in size (average decrease of 2.5 ± 2.4 cm in diameter); additionally, 95.7% (66/69) had a decrease in vascularity after one episode of TAE, with three hypervascular lesions post-TAE (50–75%). Durable radiological success was 84.1% (58/69), where seven lesions had an increase in vascularity (of which only two required repeat embolization), one lesion had no change in hypervascularity as mentioned previously, and an additional three lesions had a small increase in diameter and volume with a decrease in vascularity. Two patients required a third embolization. Three patients underwent partial nephrectomy; one had surgery after a repeat embolization, while the other two proceeded to surgery after initial failed embolization.

### 3.4. Logistic Regression Model

A multivariate logistic regression analysis was conducted to identify predictors of hemorrhage (Table 5). Diameter pre-embolization was the only statistically significant predictor of hemorrhage (*p* = 0.011). Of note, neither tumor vascularity (*p* = 0.709), etiology (*p* = 0.516), nor the presence of an aneurysm or arteriovenous malformation (*p* = 0.735) were significant predictors of hemorrhage. The smallest tumor that was complicated by hemorrhage was 4.1 cm in diameter and 22 mL in volume. Cut-point thresholds were generated from these logistic models to predict a diameter above which bleeding risk increases. A model using a single maximum diameter identified 6.8 cm as the threshold to be the critical value. Using these models and threshold diameters, hemorrhage could be predicted with a sensitivity and specificity of 84.6% and 71.3%, respectively.

An additional logistic regression model was developed to identify predictors of repeat TAE. Tumor volume after initial embolization was the only significant predictor for repeat hemorrhage (*p* = 0.001). A cut-point threshold of 248.35 mL was identified as the critical value, with a sensitivity of 55.6% and specificity of 73.7%.

### 3.5. Technical Failures and Complications

Five technical failures were identified in this study. In four cases, the vascular supply to the AML was small and tortuous, and the distal branch feeding the AML could not be catheterized. In one case, the final angiogram revealed mild residual tumor vascularity arising from inferior perforating branches, indicating incomplete embolization.

Six complications were reported in this study, four of which were minor (SIR Severity Grade I). One patient experienced a radiation burn to the flank on the affected side, which was evaluated by dermatology. The patient had a fluoroscopy time of 35.5 min, reference air kerma of 2447 mGy, and kerma area product of 740,225 mGy-cm^2^. The patient was treated with steroid cream and had no further complications. One patient developed right-sided flank pain with cramping that was treated with a week of opioid medication. Another patient developed moderate hydronephrosis with initial concern for ischemic injury to the proximal ureter; however, no change in GFR was noted, and no intervention was performed. In another case, three small, peripheral wedge-shaped defects were noted in the lower, mid, and upper poles of the renal parenchyma, likely due to non-target embolization, though renal function was preserved. Two major complications were observed. One patient experienced brief asystole after alcohol embolization, requiring IV atropine and admission for telemetry monitoring. The patient’s heart rate recovered to baseline, and they were discharged the next day (SIR Severity Grade II). Another patient developed persistent vision loss the morning after embolization, likely due to discontinuation of anticoagulation therapy five days prior to TAE. MRI revealed a left PCA territory infarct, and vision loss persisted (SIR Severity Grade IV).

## 4. Discussion

Angiomyolipomas are benign tumors that are often incidentally detected. Though they rarely pose a diagnostic dilemma, management guidelines are less established. This study presents a single-center experience in the embolization of renal AMLs over a 14-year period. Here, we evaluate the clinical and radiologic outcomes of TAE for AMLs, with a focus on identifying predictors of hemorrhage and the need for repeat intervention. Our findings reinforce the growing role of TAE as a nephron-sparing, minimally invasive therapy for both symptomatic and prophylactic management of AMLs. We found that pre-embolization tumor diameter was the only significant predictor of hemorrhage. Logistic regression demonstrated that a threshold diameter of 6.8 cm was associated with an increased bleeding risk (sensitivity 84.6%), and the smallest tumor that bled was 4.1 cm. Post-embolization tumor volume was the only significant predictor for repeat TAE, with a threshold of 248 mL.

Historically, treatment for renal AMLs has often been recommended once tumors exceed 4 cm in size—a threshold first proposed in a 1986 study by Oesterling et al., which included only 13 patients and predated the widespread use of arterial embolization [10]. In recent years, this traditional cutoff has been increasingly challenged. The European Association of Urology (EAU) now acknowledges that a definitive size threshold has not been established and cautions against using 4 cm as an automatic trigger for intervention [20]. Similarly, the Canadian Urological Association (CUA) notes that the 4 cm guideline lacks robust supporting evidence [7]. Consequently, several studies have aimed to refine this criterion. Kuusk et al. proposed a 6 cm threshold, observing that 75% of hemorrhagic events occurred in tumors exceeding this size [21]. Another study found that intervening at 4 cm could result in overtreatment in as many as 65% of cases [22]. In our cohort, only 12.9% of AMLs larger than 4 cm experienced hemorrhage, with most bleeding events occurring in tumors greater than 8 cm. We identified an optimal size threshold of 6.8 cm, corresponding to a sensitivity of 84.6% and a specificity of 71.3%. While previous models using a 4 cm cutoff achieved a sensitivity of 100%, they demonstrated substantially lower specificity of 38% [8]. Although our proposed threshold sacrifices some sensitivity, it nearly doubles specificity, allowing for more selective intervention. Our model allows for a less restrictive threshold for intervention on size alone, with more room for active surveillance and integration of additional factors that are not simply captured by a single measurement, such as degree of symptoms, personal or family history, and patient preference.

Prior studies have identified increased vascular enhancement, presence of aneurysmal vessels, and underlying etiologies such as TSC or LAM as significant risk factors for hemorrhage [5,6,8]. Yamakado et al. found the presence of intralesional aneurysms ≥5 mm to be a significant indicator of imminent AML rupture [8]. Lin et al. and Kato et al. noted that approximately 11.4% of embolized AMLs continued to grow post-TAE, particularly those with low angiomyogenic components and limited vascular supply, suggesting that baseline vascularity may also influence tumor response to treatment rather than just bleeding risk [23,24]. In our study, we did not observe a statistically significant association between vascularity, etiology (TSC/LAM), or the presence of aneurysms and hemorrhagic presentation. This discrepancy may be due in part to limited statistical power, as only 29 patients in our cohort had identifiable aneurysms, and 15 patients had a known diagnosis of TSC or LAM. Furthermore, hemorrhagic events were observed in only 14 tumors (20%), reducing the likelihood of detecting robust associations in subgroup analyses. These findings underscore the need for larger, multicenter studies to clarify the predictive value of these features and guide risk stratification.

TAE proved to be highly effective in reducing both tumor size and vascularity with a clinical success rate of 87% and an overall radiologic success rate near 100%. There was a significant difference between pre-embolization and post-embolization vascularity, diameter, and volume. No significant differences in post-embolization tumor size or vascularity were observed between hemorrhagic and non-hemorrhagic groups, suggesting that TAE may achieve comparable radiological outcomes regardless of initial clinical presentation. There is currently no consensus on the preferred use of an embolization agent. Some studies have recommended coils and microparticles as permanent embolic agents whereas other studies have recommended N-butyl cyanoacrylate glue or Onyx, as these embolics do not depend on coagulation to be effective and can act on various vascular levels [25,26,27]. Lee et al. found that ethanol was favorable for TAE as it causes irreversible endothelial damage and tumor death, yet the risk for nontarget embolization is higher [17]. A study of TAE using ethanol found a mean size reduction of 45.7% in a follow-up period of 37.7 months, but they also noted that 18.5% of patients suffered from post-embolization syndrome [28]. Embolization of AML at our institution was performed with a wide variety of embolic materials (Table 2). Consistent with previous studies, our analysis found no significant differences in tumor diameter or volume reduction across different embolic agents, suggesting that the choice of embolic agent may not substantially impact response in AML treatment.

Nine tumors (13%) required repeat TAE, a similar proportion to prior studies. A systematic review reported a repeat TAE rate of 20.9%, despite a shorter mean follow-up of 39 months, and another long-term study cited re-embolization rates as high as 41.1% over 10 years [29,30]. Few studies have investigated specific predictors for requiring re-intervention. In our analysis, post-embolization tumor volume emerged as a statistically significant predictor of repeat embolization. We identified a threshold volume of 248 mL, which demonstrated high sensitivity (73.7%) but limited specificity (55.6%). This suggests that smaller residual volumes are generally associated with a lower likelihood of recurrence or treatment failure, whereas larger post-embolization volumes may warrant closer monitoring or consideration for additional therapy. However, these findings should be interpreted with caution, as our study may be underpowered to robustly determine predictors of re-intervention, given that only nine patients underwent repeat TAE.

In our series, three patients ultimately required partial nephrectomy—one following repeat TAE and two after initial embolization. These cases involved particularly large AMLs, each exceeding 12 cm in diameter, which showed only modest reductions in size (mean decrease of 3.1 cm) after initial embolization. Six technical failures were recorded in this study, all of which stemmed from challenging vascular anatomy or incomplete embolization. Our complication rate was 7.2%, comparable to others in the literature (2.90–14.29%) [30,31,32,33]. Complications of post-embolization syndrome, renal abscesses, renal artery dissections, and lipiduria-associated UTI post-TAE have been previously documented [31,32,34]. We had two major complications: one patient with brief asystole after alcohol embolization that required medical treatment and overnight monitoring, and another patient who suffered a stroke after holding anticoagulation longer than advised [35].

The authors acknowledge several limitations inherent to this study. First, as a retrospective, single-center analysis with 66 patients, the study is subject to selection and information biases that may limit generalizability. The small number of hemorrhagic events (n = 14) and re-interventions (n = 9) may have reduced the statistical power to detect certain associations, particularly for less common variables such as TSC/LAM or intratumoral aneurysms. Additionally, in cases presenting acutely with hemorrhage and no prior imaging available, accurate pre-embolization measurements may be affected by the presence of intra-tumoral hematoma, potentially leading to overestimation of true AML size. Another limitation involves the assessment of tumor vascularity, which was categorized semi-quantitatively based on expert interpretation of both contrast-enhanced and non-enhanced imaging. This subjective method, though practical in a retrospective setting, may have introduced variability. Future investigations using standardized imaging protocols and quantitative volumetric or perfusion-based assessments would provide a more objective measure of tumor composition and vascularity. Lastly, variability in follow-up duration may have impacted the ability to fully capture long-term outcomes such as delayed hemorrhage, recurrence, or renal function decline. Prospective, multicenter studies with larger cohorts and standardized protocols are needed to validate and build upon these findings.

In conclusion, our study supports the use of TAE as a safe and effective treatment for AMLs and identifies pre-treatment tumor diameter and post-treatment volume as important predictors of hemorrhage and re-intervention, respectively. A size threshold of 6.8 cm and a post-embolization volume threshold of 248 mL may serve as valuable clinical benchmarks to guide initial treatment decisions and follow-up strategies.

## Figures and Tables

**Table 1:** Patient and AML characteristics.

**Patient Demographics**	
Age (years)	55 ± 16
Sex (female)	52 (79)
Race	
White	29 (44)
Black	3 (4.5)
Hispanic	11 (17)
Asian	16 (24)
Unknown	7 (11)
**Angiomyolipoma Characteristics**	
Exophytic	61 (88)
Aneurysm	29 (42)
Presentation	
Hemorrhage	8 (12)
Hematuria	4 (5.8)
Pain	8 (12)
Known LAM/TSC	15 (22)
Incidental	34 (49)
Etiology: Sporadic	54 (78)
Indication	
Hemorrhage	14 (20)
Symptomatic	13 (19)
Prophylactic	42 (61)

Values are presented as mean ± standard deviation or number (%).

**Table 2:** Embolic agent(s) used for AML.

Alcohol (with or without lipiodol)	25 (3 with supplementary coils)
Particles	17 (6 with supplementary coils)
Alcohol and Particles	13 (3 with supplementary coils)
Alcohol and Liquid Embolic	12 (0 with supplementary coils)
Liquid Embolic	2 (0 with supplementary coils)

Values are presented as raw numbers with the number of supplementary coils. Liquid embolic agents include: nBCA glue, Onyx, Obsidio, and Avitene.

**Table 3:** AML characteristic differences between pre- and post-embolization.

	Pre-Embolization	Post-Embolization	Significance
Vascularity			<0.001 *
<25% quartile	12 (17)	53 (77)	
25–50% quartile	18 (26)	13 (19)	
50–75% quartile	18 (26)	3 (4.3)	
>75% quartile	21 (30)	0 (0)	
Diameter (cm)	7.7 ± 3.8	5.1 ± 3.1	<0.001 *
Volume (mL)	209 ± 314	82 ± 144	<0.001 *

Values are presented as mean ± standard deviation or number (%). Asterisk (*) signifies *p* < 0.05.

**Table 4:** Difference in AML characteristics between hemorrhage and non-hemorrhage.

Characteristics	Hemorrhage (n = 14)	Non-Hemorrhage (n = 55)	Significance
**Size Threshold**			0.085
≥4.0 cm	14 (100)	48 (87)	
≥6.0 cm	10 (71)	26 (47)	
≥8.0 cm	8 (57)	16 (29)	
≥10.0 cm	5 (36)	9 (16)	
**Vascularity**			
Pre-Embolization			
0–25% quartile	1 (7.1)	11 (20)	0.987
26–50% quartile	5 (36)	13 (24)	
51–75% quartile	5 (36)	13 (24)	
76–100% quartile	3 (21)	18 (33)	
Post-Embolization			
0–25% quartile	12 (86)	41 (75)	0.506
26–50% quartile	1 (7.1)	11 (20)	
51–75% quartile	1 (7.1)	3 (5.5)	
76–100% quartile	0 (0)	0 (0)	
**Diameter (cm)**			
Before Embolization	8.4 ± 4.4	7.2 ± 3.5	0.085
After Embolization	6.0 ± 4.0	4.8 ± 2.9	0.516
Overall Change	−3.2 ± 1.7	−2.3 ± 2.5	0.034*
**Volume (mL)**			
Before Embolization	414 ± 520	156 ± 214	0.053
After Embolization	163 ± 237	63 ± 106	0.606
Overall Change	−221 ± 251	−89 ± 140	0.016*

Values are presented as mean ± standard deviation. Asterisk (*) signifies *p* < 0.05.

**Table 5:** A. Multivariate logistic regression analysis of predictors of hemorrhage. B. Multivariate logistic regression analysis of predictors of repeat hemorrhage.

Predictors	Odds Ratio	95% CI	*p*-Value
A: Multivariate logistic regression analysis predictors for hemorrhage			
Age	0.995	0.988–1.003	0.298
Gender	0.804	0.576–1.122	0.214
Etiology	1.101	0.780–1.553	0.516
Vascular malformation	0.959	0.712–1.292	0.735
Tumor vascularity (quartile)	0.977	0.851–1.122	0.709
Diameter (pre-embolization)	0.994	0.993–0.995	0.011*
Volume (pre-embolization)	1.002	1.001–1.003	0.247
B: Multivariate logistic regression analysis predictors for repeat hemorrhage			
Age	1.017	0.000–0.624	0.488
Gender	0.424	0.961–1.085	0.568
Etiology	1.894	0.020–3.215	0.469
Vascular malformation	1.338	0.242–6.989	0.635
Tumor vascularity (post-embolization)	1.587	0.413–5.772	0.477
Diameter (post-embolization)	0.994	0.958–1.031	0.733
Volume (post-embolization)	1.004	1.004–1.004	0.001*

CI = confidence interval. Asterisk (*) signifies *p* < 0.05.

## Data Availability

The datasets generated and/or analyzed during the current study are available from the corresponding author on request.
